# Effects of phytogenic feed additives on cellular oxidative stress and inflammatory reactions in intestinal porcine epithelial cells^1^

**DOI:** 10.1093/jas/sky263

**Published:** 2018-07-05

**Authors:** Theresa Kaschubek, Elisabeth Mayer, Sophia Rzesnik, Bertrand Grenier, Diana Bachinger, Carina Schieder, Jürgen König, Klaus Teichmann

**Affiliations:** 1 BIOMIN Research Center, Tulln an der Donau, Austria; 2 Department of Nutritional Science, University of Vienna, Vienna, Austria; 3 BIOMIN Holding GmbH, Getzersdorf, Austria

**Keywords:** antibiotic growth promoter, gut health, intestinal inflammation, intestinal porcine epithelial cells, oxidative stress, plant-derived substances

## Abstract

Due to increasing concerns about the use of antibiotic growth promoters (**AGP**) in livestock production and their complete ban in the European Union in 2006, suitable alternatives are urgently needed. Among others, anti-inflammatory activities of AGP are discussed as their putative mode of action. As numerous phytochemicals are known to modulate the cellular antioxidant capacity and immune response, we studied the antioxidative and anti-inflammatory properties of a phytogenic (plant-derived) feed additive (**PFA**) in intestinal porcine epithelial cells (**IPEC-J2**). The effects of the PFA were compared with those of selected phytogenic ingredients (grape seed extract [**GRS**], licorice extract [**LIC**], menthol [**MENT**], methyl salicylate [**MES**], oak bark extract [**OAK**], oregano essential oil [**ORE**], and a plant powder mix [**PLA**]), and with the effects of the AGP tylosin (**TYL**). Oxidative or inflammatory stress was induced by stimulating IPEC-J2 with hydrogen peroxide (**H**_**2**_**O**_**2**_; 0.5 m*M*) or tumor necrosis factor alpha (**TNF-α**; 10 ng/mL), respectively. The antioxidative effects of feed additives were assessed with a reactive oxygen species (**ROS**)-sensitive probe and by measuring the expression of 6 antioxidative target genes via quantitative real-time PCR (**RT-qPCR**). Anti-inflammatory potential was analyzed using a nuclear factor kappa-light-chain-enhancer of activated B cells (**NF-κB**) reporter gene assay. Moreover, the expression levels of 6 NF-κB target genes were measured using RT-qPCR analysis, and the release of IL-6 was analyzed via ELISA. Significant decreases in cellular ROS upon H_2_O_2_ treatment were observed for the PFA (*P* < 0.001), LIC (*P* < 0.001), ORE (*P* < 0.05), and GRS (*P* < 0.01). No significant changes in the expression of antioxidative genes were found. NF-κB activation upon TNF-α treatment was significantly inhibited by the PFA (*P* < 0.05) and by ORE (*P* < 0.001). Moreover, the PFA and ORE significantly reduced the gene expression of IL-6 (*P* < 0.001), IL-8 (*P* < 0.001), and C-C motif chemokine ligand 2 (**CCL2**; *P* < 0.05), as well as the release of IL-6 (*P* < 0.05). The other phytogenic compounds as well as the AGP TYL did not significantly affect any of the inflammatory parameters. In summary, we revealed the antioxidative properties of the PFA, LIC, ORE, and GRS, as well as anti-inflammatory properties of the PFA and ORE in IPEC-J2, providing a better understanding of the mode of action of this PFA under our experimental conditions.

## INTRODUCTION

Gastrointestinal health in pigs is directly related to animal productivity and is a major concern for the modern swine industry. Exposure of pigs to various stressors (e.g., early-weaning, diet transition, or pathogens) is associated with a disturbance of the oxidative balance and a tremendous release of immunological agents in the intestine, which contribute to gastrointestinal disorders and poor animal health (Pié et al., 2004; Dong, 2007; Yin et al., 2014).

Antibiotic growth promoters (**AGP**) have been used to control gastrointestinal dysfunction and to improve growth performance. Since the ban of AGP in the European Union in 2006, phytogenic (plant-derived) feed additives (**PFA**) have attracted considerable attention in livestock industry (Windisch et al., 2008). Numerous studies have reported beneficial effects of PFA on growth performance and feed conversion, in swine and poultry (Hashemi and Davoodi, 2011). It is hypothesized that modulation of both the intestinal immune response and cellular antioxidant capacity by plant-derived substances is relevant for their activity (Mueller et al., 2012; Liu et al., 2014). However, to optimize the use of PFA in animal production and implement them as viable alternatives to AGP, the field will need a more detailed understanding of their mechanism of action, specifically, of potential effects on immune regulation and redox homeostasis.

Therefore, this study aimed to elucidate the anti-inflammatory and antioxidative activities of a complex PFA and selected phytogenic ingredients in intestinal porcine epithelial cells (**IPEC-J2**). IPEC-J2 show strong similarities to the original tissue and are a well-accepted in vitro model for studies on the intestine (Schierack et al., 2006). Moreover, the anti-inflammatory effects of the PFA were compared with those of tylosin (**TYL**), which was formerly used as an AGP in the EU and is still used in other regions of the world.

## MATERIALS AND METHODS

### Phytogenic Substances and an Antibiotic Growth Promoter

The PFA Digestarom DC (**D-DC**) (BIOMIN Phytogenics GmbH, Stadtoldendorf, Germany) comprises a complex mixture of single phytogenic components (Table 1). The PFA and selected ingredients were tested individually in this study. Grape seed (**GRS**), licorice (**LIC**), and oak bark (**OAK**) extracts, a plant powder mix (**PLA**), and the essential oil of oregano (**ORE**) were provided by BIOMIN Phytogenics GmbH (Stadtoldendorf, Germany), menthol (**MENT**) was purchased from Sigma-Aldrich (St. Louis, Missouri, USA), and methyl salicylate (**MES**) and tylosin tartrate 95% were obtained from Alfa Aesar (Haverhill, Massachusetts, USA).

**Table 1.** Selected components of Digestarom DC (D-DC) and applied test concentrations

Item	Abbreviation	Characterization	Test concentration, µg/mL
Grape seed extract	GRS	Water extract of *Vitis vinifera* seeds; total phenols ≥ 40%	1, 2, 4
Licorice extract	LIC	Water extract of *Glycyrrhiza glabra* roots; glycyrrhizic acid > 5.5%	15, 30, 60
L-menthol	MENT	Natural, from *Mentha arvensis* aerial parts; Purity > 99%	3.125, 6.25, 12.5
Methyl salicylate	MES	Purity > 99%	1, 3.125, 6.25
Oak bark extract	OAK	Ethanol extract of *Quercus robur* bark; tannins 4.9%, calculated as pyrogallol	1, 2, 4
Oregano	ORE	Essential oil of *Origanum vulgare* aerial parts; carvacrol 60–75%	25, 50, 100
Plant powder mix	PLA	Dried and ground *Cinnamomum verum* bark, *Gentiana lutea* root, and *Angelica archangelica* root	10, 20, 40

Stock solutions of powdered samples were prepared by adding 70% ethanol and shaking for 1 h. Liquid samples were dissolved in absolute ethanol and TYL was solubilized in sterile water. Stock solutions were sterile filtered through a 0.2-µm filter (Sarstedt GmbH, Biedermannsdorf, Austria) before they were used for preparation of the required test solutions. The effects of the solvents and carriers contained in D-DC were tested in corresponding concentrations in the respective assays, and the concentrations of test substances were chosen so that effects of solvents and carriers were excluded.

Stock solutions were diluted in complete growth medium for the cell viability assay, nuclear factor kappa-light-chain-enhancer of activated B cells (**NF-κB**) activation assay, ELISA, and quantitative real-time PCR (**RT-qPCR**) analysis. For oxidative stress analysis, the stock solutions were diluted in Hanks’ Balanced Salt Solution (**HBSS**) (Gibco, Life Technologies, Carlsbad, California, USA), to exclude potential extracellular side reactions between the reactive oxygen species (**ROS**)-sensitive probe and the complete growth medium. In the cell viability assay, oxidative stress assay, and NF-κB activation assay, 3 different concentrations of each test substance were studied, which correspond to inclusion rates applied in feed. D-DC was tested at 150, 300, and 600 µg of product per mL of complete growth medium, and the plant extracts, PLA, and essential oils were tested as shown in Table 1. TYL was tested at 100 µg/mL for anti-inflammatory activity. For ELISA and RT-qPCR analyses, one concentration of each test substance was selected, which was chosen on the basis of anti-inflammatory or antioxidative activity in the previous screening assays.

### Cell Culture

IPEC-J2 (ACC 701; Leibniz Institute DSMZ-German Collection of Microorganisms and Cell Culture, Braunschweig, Germany) were cultured in complete growth medium, consisting of Dulbecco’s modified Eagle’s medium/Ham’s F-12 (1:1) without glutamine (Biochrom AG, Berlin, Germany), supplemented with 5% fetal bovine serum, 2.5 m*M* GlutaMAX, 1% insulin-transferrin-selenium (all from Gibco Life Technologies, Carlsbad, California, USA), 5 ng/mL epidermal growth factor (Corning, New York, USA), 16 m*M* HEPES, and 1% penicillin-streptomycin (both from Sigma-Aldrich, St. Louis, Missouri, USA), at 39 °C and 5% CO_2_ under humidified conditions. Cells were routinely maintained in 150 cm^2^ cell culture flasks (Starlab International GmbH, Hamburg, Germany) and were split every 3 to 4 d after reaching confluence for a maximum of 15 passages. Cells were regularly checked for mycoplasma contamination via PCR analysis (Venor GeM Mycoplasma Detection Kit, PCR based, Minerva Biolabs, Berlin, Germany).

### Cell Viability Assay

The neutral red (**NR**) cell viability assay (Aniara Diagnostica, West Chester, Ohio, USA), which targets lysosomal activity, was performed to study the cytotoxic influence of the phytogenic test substances on IPEC-J2 and to define nontoxic test concentrations for further experiments. IPEC-J2 were seeded at a density of 3 × 10^4^ cells/well in 96-well flat-bottom plates (Eppendorf, Hamburg, Germany) and cultured for 24 h to reach approximately 100% confluence. The supernatants were discarded and the cells were treated with 200 µL of either complete growth medium (cell control [**CC**]), various concentrations of the phytogenic substances, or 100 µg/mL TYL for 24 h. On the next day, the NR cell viability assay was performed according to the manufacturer’s instructions. Briefly, cells were washed and incubated with a 1:100 NR solution (diluted in complete growth medium) for 3 h, allowing accumulation of the dye in the lysosomes. Subsequently, the cells were fixed for 1 min, the incorporated dye was dissolved, and the absorbance was measured at 540 nm with a reference wavelength of 690 nm.

### Oxidative Stress Assay

The potential of phytogenic substances to reduce intracellular oxidative stress was assessed using the ROS-sensitive probe 2′,7′-dichlorodihydrofluorescein diacetate (**DCFH-DA**) (Sigma-Aldrich, St. Louis, Missouri, USA). Twenty-four hours after IPEC-J2 were seeded in white 96-well flat-bottom plates (Corning, New York, USA) at 3 × 10^4^ cells/well, the confluent cell layer was washed with HBSS (Gibco, Life Technologies, Carlsbad, California, USA) and was further exposed to 40 µ*M* DCFH-DA for 1 h. Subsequently, the cells were washed with HBSS 3 times and incubated with 200 µL of HBSS (CC), 200 µL of 1 m*M* gallic acid (Sigma-Aldrich, St. Louis, Missouri, USA) used as assay control, or 200 µL of the phytogenic test substances for 1 h. The IPEC-J2 were then washed with HBSS, and oxidative stress was induced by stimulation with 0.5 m*M* hydrogen peroxide (**H**_**2**_**O**_**2**_) (Merck Millipore, Billerica, Massachusetts, USA) for another 1 h. Fluorescence was measured at an excitation wavelength of 485 nm and an emission wavelength of 528 nm. Data were expressed in relative fluorescence units compared with H_2_O_2_-stimulated cells (stimulated control (**SC**)), which were defined as 100%.

### NF-κB Reporter Gene Assay

The effects of phytogenic substances and TYL on tumor necrosis factor alpha (**TNF-α**) (R&D Systems, Minneapolis, Minnesota, USA)-induced activation of the inflammatory transcription factor NF-κB were examined using an NF-κB reporter gene assay (BPS Bioscience, San Diego, California, USA). To reach approximately 70% confluence, which is decisive for successful transfection, IPEC-J2 were seeded at 6 × 10^3^ cells/well in white 96-well flat-bottom plates, 24 h before transfection. The confluence state of the cells was checked under the microscope before performing the assay. According to the manufacturer’s protocol, IPEC-J2 were transiently transfected with the NF-κB luciferase reporter vector using Lipofectamine 2000 transfection reagent (Invitrogen, Life Technologies, Carlsbad, California, USA). After 24 h of transfection, the transfection reagent was removed, and the cells were exposed to 200 µL of complete growth medium (CC) or 200 µL of the test substances for another 24 h. The NF-κB inhibitor BAY 11–7085 (5 µ*M*) (Cayman Chemical, Ann Arbor, Michigan, USA), which was used as the assay control, was added 1 h before NF-κB was activated by stimulation of the cells with 10 ng/mL TNF-α for the recommended incubation time of approximately 5 h. The effects of the test samples were additionally analyzed without TNF-α stimulation to determine potential activation of NF-κB by the test substances alone compared with the untreated CC. Luciferase activity was measured by the Dual-Glo Luciferase Assay (Promega, Madison, Wisconsin, USA) and relative luminescence values were calculated, in comparison with the TNF-α-stimulated cells, which were defined as 100%.

### Gene Expression Analysis

The expression levels of 2 housekeeping genes, 6 inflammation-related target genes, and 6 genes belonging to the cellular antioxidative response system were analyzed at the messenger RNA (**mRNA**) level via RT-qPCR (Table 2). For gene expression analysis, IPEC-J2 were seeded at 2.2 × 10^5^ cells/well in 12-well plates and were grown for 24 h. The next day, either 1 mL of complete growth medium (CC) or 1 mL of a test product was added to the cells for 1 h. Expression of inflammatory genes was then induced by stimulation of IPEC-J2 with 10 ng/mL TNF-α, whereas expression of antioxidative target genes was activated by treatment with 0.5 m*M* H_2_O_2._ After 1 h of stimulation with 1 of the 2 stimuli, the supernatants were removed and 500 µL of RNA*later* Stabilization Solution (Invitrogen, Life Technologies, Carlsbad, California, USA) was added to protect the cellular RNA. Samples were stored at −80 °C until further processing.

**Table 2.** List of housekeeping genes, inflammation-related target genes, and antioxidative target genes analyzed in this study

Gene symbol	Official full name
Housekeeping genes	
GAPDH	Glyceraldehyde-3-phosphate dehydrogenase
ACTB	Beta-actin
Inflammatory target genes	
IL-6	Interleukin 6
CXCL8	C-X-C motif chemokine ligand 8, also known as interleukin 8
TNF-α	Tumor necrosis factor alpha
IL-1β	Interleukin 1 beta
CCL2	C-C motif chemokine ligand 2
IL-10	Interleukin 10
Antioxidative target genes	
SOD1	Superoxide dismutase 1
HMOX1	Heme oxygenase 1
NQO1	NAD(P)H quinone dehydrogenase 1
PRDX6	Peroxiredoxin 6
GPX2 (isozyme of the glutathione peroxidase family that is predominantly expressed in the gastrointestinal tract)	Glutathione peroxidase 2
CAT	Catalase

Total RNA was isolated using the RNeasy Mini Kit (Qiagen GmbH, Hilden, Germany) as described in the manufacturer’s instructions. Isolated RNA samples were shipped on dry ice to Qiagen GmbH (Hilden, Germany), where the measurement of concentration and quality of RNA (via Nano Drop spectrophotometer and RNA integrity number measurement), cDNA synthesis, and RT-qPCR were conducted. The threshold cycle (**C**_**t**_) values of the genes of interest and the 2 housekeeping genes were provided by Qiagen and were used for data analysis. The 2^ΔΔCt^ method was used for data evaluation. Therefore, the average C_t_ values of all replicates of each treatment group were assessed. The ΔC_t_ values were calculated by subtracting the mean C_t_ values for the target genes from the mean C_t_ values for the 2 housekeeping genes. The ΔΔC_t_ values, expressing the differences in ΔC_t_ between the experimental treatment groups and the untreated CC, were further used for statistical evaluation. The results for the test substances were compared with those for TNF-α stimulated cells and are illustrated as ΔΔC_t_ values, which correspond to the binary logarithm (**log**_**2**_) of the fold change (fold change = 2^ΔΔCt^ value). Cutoff values of <−1.5 or >1.5 were used to identify relevant changes in gene expression.

### Cytokine Detection by ELISA

The porcine IL-6 Quantikine ELISA Kit (R&D Systems, Minneapolis, Minnesota, USA) was used to determine regulation of the inflammation-related cytokine IL-6 by test substances in IPEC-J2. The cells were seeded in 12-well plates at 2.2 × 10^5^ cells/well (Eppendorf, Hamburg, Germany) for 24 h, preincubated with 1 mL of the test substances for 1 h, and subjected to inflammatory stimulation with 10 ng/mL TNF-α for 5 h. After that cell culture supernatants were collected and assayed undiluted according to the manufacturer’s protocol.

### Statistical Analysis

Data from all experiments were expressed as the means± SEM, and statistical analysis was performed using absolute data with the statistical software GraphPad Prism 5 (Version 5.0, GraphPad Software, Inc., La Jolla, California, USA) for all assays except the NF-κB reporter gene assay, which was analyzed with IBM SPSS Statistics (Version 19.0, IBM corp., New York, USA). Data were checked for normality via the Kolmogorov–Smirnov normality test. If the data passed the normality test, ANOVA or two-way ANOVA in the case of the NF-κB reporter gene assay, and Dunnett’s test were performed to identify significant differences between the mean values of the experimental groups and those of the control group. Data that were not normally distributed were further analyzed via the nonparametric Kruskal–Wallis test. Differences were considered statistically significant if their *P*-values were <0.05.

## RESULTS

### Cytotoxic Effects of Phytogenic Substances and Tylosin on IPEC-J2

**Figure 1.**
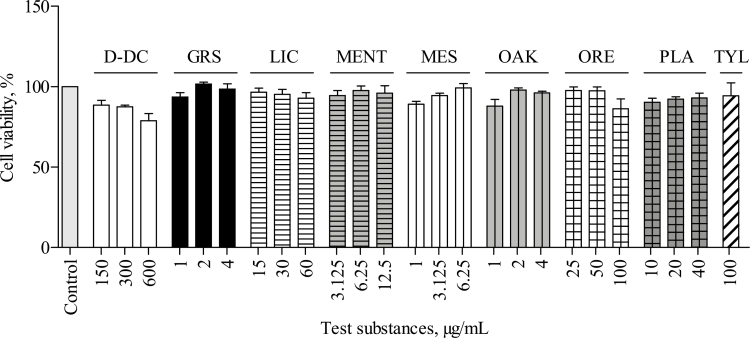
Effect of Digestarom DC (D-DC), grape seed extract (GRS), licorice extract (LIC), menthol (MENT), methyl salicylate (MES), oak bark extract (OAK), oregano essential oil (ORE), plant powder mix (PLA), and tylosin (TYL) on cell viability of intestinal porcine epithelial cells (IPEC-J2). IPEC-J2 were incubated with the test substances for 24 h and cell viability was assessed via the neutral red cell viability assay (NR assay). Relative viability in comparison to the untreated control, which was set to 100%, is displayed. Data represent the mean values and SEM of 4 independent experiments (*n* = 4). No significant differences were found.

Potential cytotoxic effects of the test substances on the viability of IPEC-J2 were assessed via the NR assay. None of the studied test substances significantly reduced the viability of the treated cells compared with that of the untreated control cells (*P* > 0.05; Figure 1).

### Influence of Phytogenic Test Substances on Oxidative Stress in IPEC-J2

**Figure 2.**
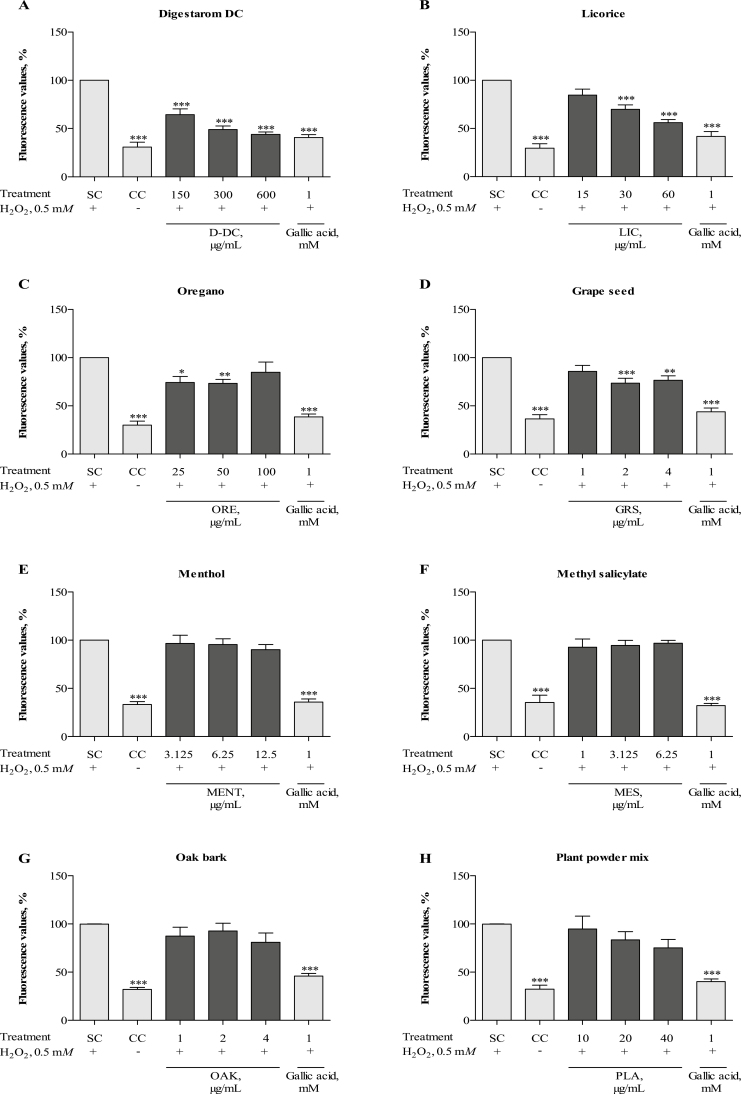
Antioxidative effect of Digestarom DC (D-DC) (A), licorice extract (LIC) (B), oregano essential oil (ORE) (C), grape seed extract (GRS) (D), menthol (MENT) (E), methyl salicylate (MES) (F), oak bark extract (OAK) (G), and the plant powder mix (PLA) (H) in intestinal porcine epithelial cells (IPEC-J2). Cells were exposed to 40 µ*M* 2′, 7′-dichlorodihydrofluorescein diacetate (DCFH-DA) for 1 h. After subsequent preincubation with the test substances for 1 h, production of reactive oxygen species (ROS) was induced by stimulation with 0.5 m*M* hydrogen peroxide (H_2_O_2_) for another 1 h. Relative fluorescence values compared with the H_2_O_2_-treated control (SC) (set to 100%) are shown. Each diagram also shows the untreated control (CC) and the effects of the assay control gallic acid. The results represent the mean and SEM of five independent experiments (*n* = 5). Significant differences are marked by asterisks (*P < 0.05; ** *P* < 0.01; *** *P* < 0.001).

Untreated control cells had significantly lower levels of cellular ROS compared with cells stimulated with 0.5 m*M* H_2_O_2_, which were defined as 100% (*P* < 0.001; Figure 2). The assay control gallic acid significantly inhibited H_2_O_2_-induced formation of ROS (*P* < 0.001). All tested concentrations of D-DC (150, 300, and 600 µg/mL) significantly decreased ROS production compared with H_2_O_2_-stimulated cells (*P* < 0.001), as did the 2 higher concentrations of LIC (30 and 60 µg/mL; *P* < 0.001) (Figure 2A and B). Treatment with 25 or 50 µg/mL of ORE significantly decreased the ROS levels upon H_2_O_2_-stimulation (*P* < 0.05 and *P* < 0.01, respectively), whereas the effect was not observed for the highest concentration (100 µg/mL) (Figure 2C). Additionally, the formation of ROS was significantly reduced by treatment with 2 and 4 µg/mL GRS (*P* < 0.001 and *P <* 0.01, respectively), however not in a dose-dependent manner (Figure 2D). MENT, MES, OAK, and the PLA did not significantly affect the presence of ROS (*P* > 0.05; Figure 2E–H).

### Influence of Phytogenic Substances and Tylosin on Gene Expression of Antioxidative Target Genes

**Figure 3.**
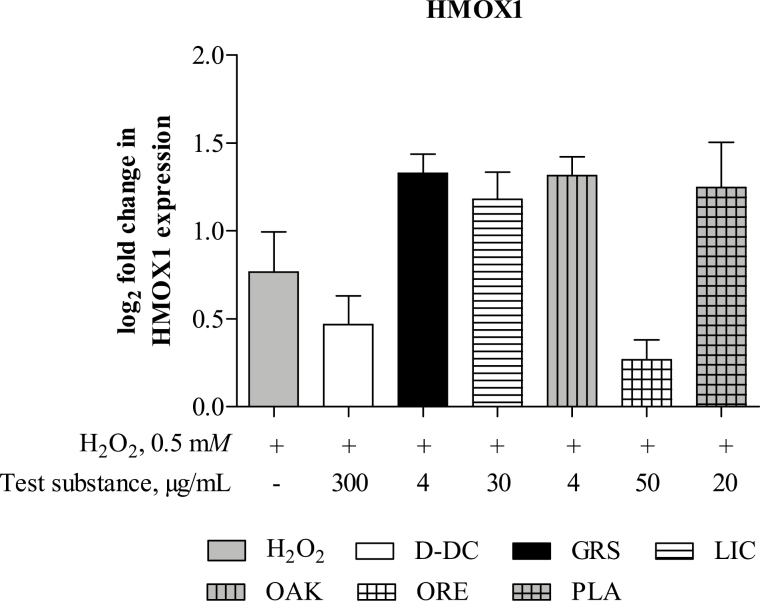
Effects of Digestarom DC (D-DC), grape seed extract (GRS), licorice extract (LIC), oak bark extract (OAK), oregano essential oil (ORE), and the plant powder mix (PLA) on gene expression of the antioxidative gene heme oxygenase 1 (HMOX1) in intestinal porcine epithelial cells (IPEC-J2). Gene expression was analyzed via RT-qPCR. Cells were preincubated with the test substances for 1 h, after which oxidative stress was induced by stimulation of the cells with 0.5 m*M* H_2_O_2_ for 1 h. The untreated cell control (CC) was set to 0 and is not shown in the figure. Cells treated with the test substances were compared with the H_2_O_2_-stimulated control, and results are presented as the binary logarithm (log_2_) of the fold increase. Data represent the mean and SEM of five independent experiments (*n* = 5). No significant differences were found.

The antioxidative target genes SOD, NQO1, PRDX6, GPX2, and CAT were highly expressed in IPEC-J2, indicated by low Ct-values of around 25; however, the fold changes between the untreated and H_2_O_2_-stimulated groups were below the defined cutoff value of >1.5 (data not shown). The expression of HMOX1 was significantly increased in H_2_O_2_-stimulated cells compared with the untreated control (*P* < 0.05; Figure 3). None of the studied test substances significantly affected the expression of HMOX1 (*P* > 0.05) in comparison to its expression in the H_2_O_2_-stimulated cells.

### Effects of Phytogenic Test Substances and Tylosin on Activation of the Inflammatory Transcription Factor NF-κB

**Figure 4.**
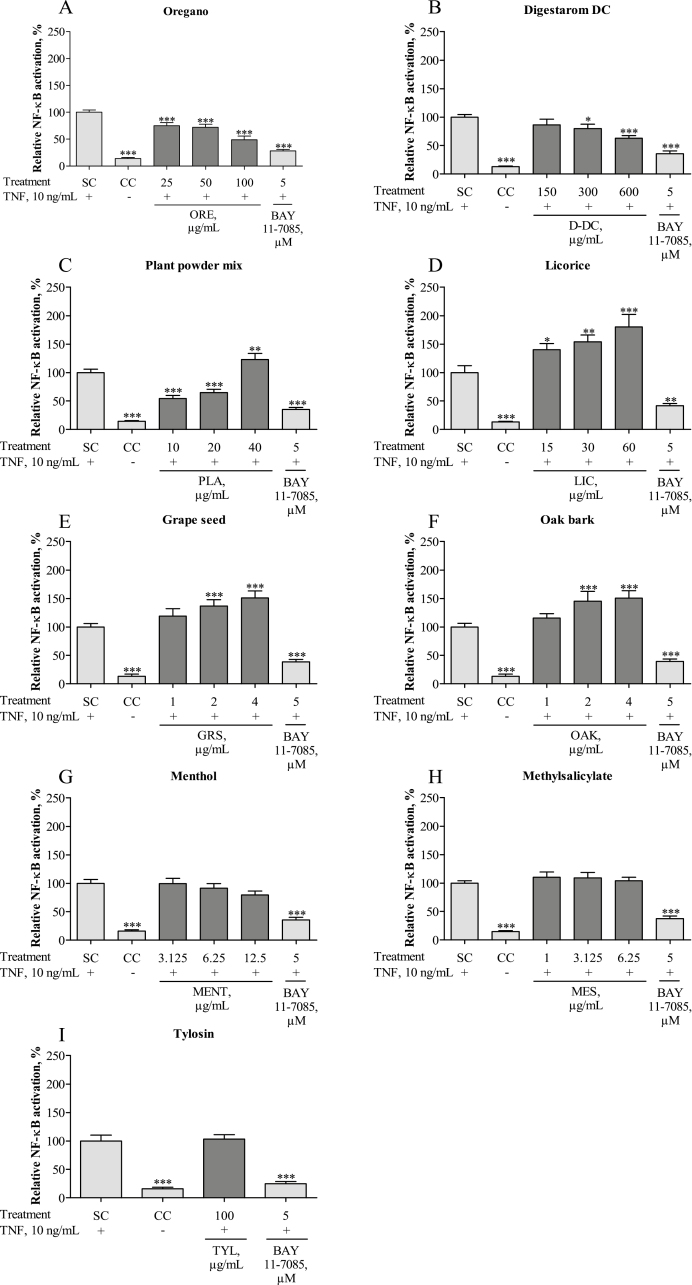
NF-κB reporter activation after inflammatory stimulation with tumor necrosis factor alpha (TNF-α) and treatment with oregano essential oil (ORE) (A), Digestarom DC (D-DC) (B), plant powder mix (PLA) (C), licorice extract (LIC) (D), grape seed extract (GRS) (E), oak bark extract (OAK) (F), menthol (MENT) (G), methyl salicylate (MES) (H), and tylosin (TYL) (I) in intestinal porcine epithelial cells (IPEC-J2). Cells were transiently transfected with the NF-κB reporter gene vector for 24 h and further incubated with the different test substances for 24 h. The NF-κB inhibitor BAY 11–7085 (5 µ*M*) was added to the cells for 1 h before NF-κB activation was induced by stimulation with 10 ng/mL TNF- α for 5 h. Luminescence was measured, and the figure shows relative NF-κB reporter activation compared with the TNF-α treated control (SC), which was set to 100%. Each diagram also shows the untreated cell control (CC) and the effects of the assay control BAY 11–7085. The results represent the mean and SEM of 5 independent experiments (*n* = 5). Significant differences are marked by asterisks (* *P* < 0.05; ** *P* < 0.01; *** *P* < 0.001).

Inflammatory stimulation by TNF-α induced significant up-regulation of NF-κB compared with the level in the untreated CC, defined as 100% NF-κB activation (*P* < 0.001) (Figure 4). The NF-κB inhibitor BAY 11–7085, used as assay control, effectively attenuated this effect, producing values similar to those of the untreated CC (*P* < 0.001). Pretreatment with ORE was able to counteract the TNF-α induced activation of NF-κB (*P* < 0.001 at all test concentrations; Figure 4A). Significant NF-κB-restricting properties were further observed for D-DC at 300 and 600 µg/mL (*P* < 0.05 and *P* < 0.001, respectively; Figure 4B). PLA caused a significant inhibition of NF-κB at the 2 lower concentrations of 10 and 20 µg/mL (*P* < 0.001), but at 40 µg/mL NF-κB activation was even higher than in TNF-α-stimulated cells (*P* < 0.01; Figure 4C). The same phenomenon has been observed when testing LIC, GRS, or OAK. Although LIC showed significant enhancement of NF-κB at all 3 test concentrations 15, 30, and 60 µg/mL (*P* < 0.05, *P* < 0.01, and *P* < 0.001, respectively; Figure 4D), GRS and OAK enhanced the amount of NF-κB at 2 and 4 µg/mL (*P* < 0.001) but not at 1 µg/mL (Figure 4E and F). Exposure of IPEC-J2 to MENT, MES, or TYL did not affect the amount of NF-κB significantly (*P* > 0.05; Figure 4G–I). None of the test substances alone caused NF-κB activation compared with the unstimulated CC (*P* > 0.05; data not shown).

### Influence of Phytogenic Substances and Tylosin on Expression of Anti-Inflammatory Target Genes

**Figure 5.**
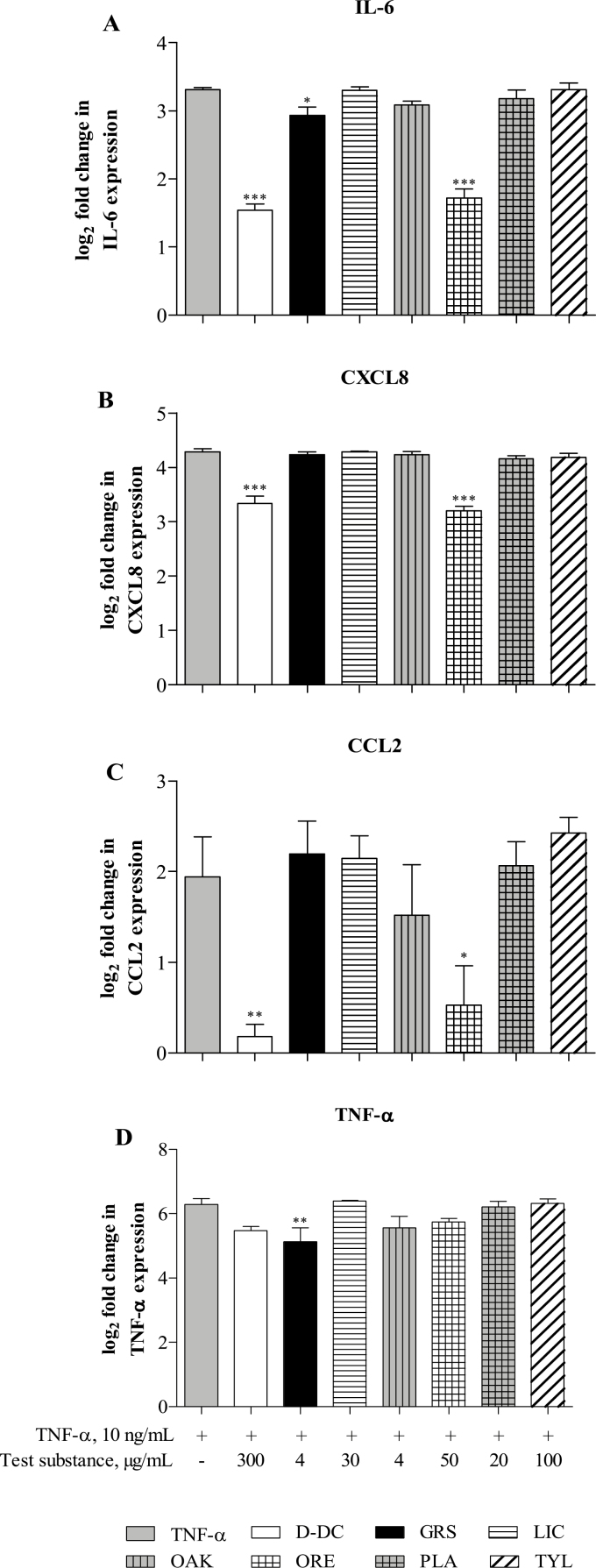
Effects of Digestarom DC (D-DC), grape seed extract (GRS), licorice extract (LIC), oak bark extract (OAK), oregano essential oil (ORE), plant powder mix (PLA), and tylosin (TYL) on expression of the inflammation-related target genes IL-6 (A), C-X-C motif chemokine ligand 8 (CXCL8) (B), tumor necrosis factor alpha (TNF-α) (C), and C-C motif chemokine ligand 2 (CCL2) (D) in intestinal porcine epithelial cells (IPEC-J2). Gene expression was analyzed via RT-qPCR. Cells were preincubated with the test substances for 1 h, after which inflammation was induced by stimulation of the cells with 10 ng/mL TNF-α for 1 h. Test substances were compared with the TNF-α stimulated control, and the results are presented as the binary logarithm (log_2_) of the fold increase. The untreated cell control (CC) was set to 0 (=log_2_(1)) and is not shown in the figure. The data represent the mean and SEM of 5 independent experiments (*n* = 5). Significant differences are marked by asterisks (*P < 0.05; ** *P* < 0.01; *** *P* < 0.001).

Induction of intestinal immune reactions by stimulation with TNF-α significantly enhanced the expression of inflammatory genes IL-6, CXCL8, TNF-α, and CCL-2 (*P* < 0.001; *P* < 0.01 for CCL2) compared with the levels in the untreated control, which were set to 0 (=log_2_(1)) and are not shown in Figure 5. D-DC and ORE significantly inhibited the expression of IL-6, CXCL8 (*P* < 0.001; Figure 5A and B), and CCL2 (*P* < 0.01 and *P* < 0.05, respectively; Figure 5C), after induction of inflammation by TNF-α. GRS significantly reduced the inflammatory parameters IL-6 and TNF-α (*P* < 0.05 and *P* < 0.01, respectively; Figure 5A and D), whereas LIC, OAK, PLA, and the AGP TYL had no impact on mRNA levels of any of the observed parameters (*P* > 0.05). The mRNA levels of the immune-related target genes IL-1β and IL-10 were detected only in very low amounts in stimulated or nonstimulated IPEC-J2. Therefore, those data were not considered for analysis and are not shown in Figure 5.

### Analysis of the Inflammatory Cytokine IL-6

**Figure 6.**
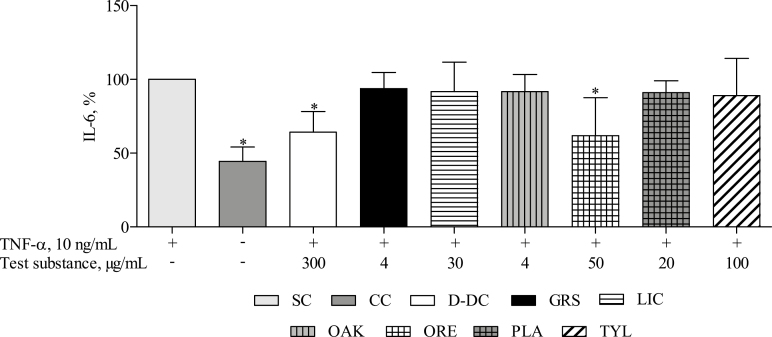
IL-6 release by intestinal porcine epithelial cells (IPEC-J2) stimulated with 10 ng/mL tumor necrosis factor alpha (TNF-α) (SC), unstimulated IPEC-J2 (CC), and cells stimulated with TNF-α in the presence of the phytogenic test substances Digestarom DC (D-DC), grape seed extract (GRS), licorice extract (LIC), oak bark extract (OAK), oregano essential oil (ORE), plant powder mix (PLA), or the antibiotic growth promoters (AGP) tylosin (TYL). Cells were preincubated with complete growth medium or the test substances for 1 h, after which the release of IL-6 was induced by stimulation of the cells with 10 ng/mL TNF-α for 5 h. The supernatants were collected and used for analysis of the amount of IL-6 on protein level via ELISA. The results represent the mean and SEM of 5 independent experiments (*n* = 5). Significant differences, compared with the TNF-α stimulated control, are marked by asterisks (*P < 0.05).

The untreated cell control showed a significantly lower basal level of IL-6 than IPEC-J2 that were treated with TNF-α (*P* < 0.05; Figure 6). Treatment with D-DC (300 µg/mL) and ORE (50 µg/mL) counteracted IL-6 release in response to TNF-α (*P* < 0.05). The other phytogenic test substances (GRS, LIC, OAK, and PLA) and TYL did not significantly influence the level of IL-6 after TNF-α stimulation (*P* > 0.05).

## DISCUSSION

Feed additives are commonly used in animal production for their sensory properties, nutritional effects, to maintain health and animal welfare, to reduce environmental consequences of animal production, to beneficially affect the characteristics of animal products, and/or to enhance growth performance of the animals (Windisch et al., 2008; Wallace et al., 2010). AGP supplementation in animal feed was widely used in livestock production in past decades; however, concerns about the development of antibiotic-resistant bacteria and antibiotic residues in animal tissues and the environment led to a complete ban of AGP in the European Union in 2006 (Niewold, 2007; Hashemi and Davoodi, 2011). Driven by this ban, as well as increasing societal concerns, the pressure to find suitable nonantibiotic replacements has increased substantially.

The use of PFA in livestock nutrition has become increasingly important (Windisch et al., 2008; Wallace et al., 2010; Gessner et al., 2017), and research on the effects of PFA on animal health and growth performance has steadily increased. Dietary PFA have improved the performance of broiler chickens and piglets in several in vivo studies (Maenner et al., 2011; Murugesan et al., 2015; Valenzuela-Grijalva et al., 2017). Modulation of the immune and oxidative defense system is regarded as one of the main functions of phytogenic compounds, responsible for improving animal health and thereby enhanced growth performance (Yang et al., 2015; Valenzuela-Grijalva et al., 2017).

In vitro studies can provide a valuable research tool to study the precise mode of action of PFA. The intestinal porcine epithelial cell line IPEC-J2 is characterized by its nontumorigenic origin and shows a high degree of resemblance to in vivo conditions of the intestinal tissue. Therefore, that cell line has become an increasingly important model in recent years (Berschneider, 1989; Schierack et al., 2006). In several studies, IPEC-J2 were successfully employed for research on intestinal inflammation and oxidative stress response (Schierack et al., 2006; Farkas et al., 2014; Palócz et al., 2016), making them an ideal cell line to study the response of intestinal epithelial cells to PFA. In the current study, we demonstrate inflammation-restricting and antioxidative properties of the complex PFA D-DC in the IPEC-J2 cell culture model and provide detailed knowledge about its mode of action. The activities of D-DC were compared with those of selected phytogenic ingredients. Moreover, we compared the anti-inflammatory properties of the plant-derived substances and the AGP TYL in IPEC-J2, to the best of our knowledge, for the first time, to investigate possible similarities in their mode of action on the gut immune system.

Oxidative stress is regarded as a result of the disruption of cellular redox homeostasis. The overproduction of ROS or inadequate clearance of ROS by the body’s antioxidant defense system can result in considerable tissue damage and even cell death (Yin et al., 2013). In intensive animal production, oxidative stress is a common challenge that animals face in various life stages. Weaning, for example, is associated with oxidative stress in piglets, which is presented by morphological alterations in various parts of the intestine, reduced activity of digestive enzymes, diarrhea, and an overall deterioration of animal health state (Zhu et al., 2012; Buchet et al., 2017). However, the use of phytogenic agents in animal nutrition seems to be highly effective against the harmful influence of oxidative stress (Settle et al., 2014; Gerasopoulos et al., 2015; Tan et al., 2015).

H_2_O_2_ is a well-known inducer of ROS and has been used previously to study oxidative stress in IPEC-J2 (Cai et al., 2013). A ROS scavenging effect of some phytochemicals (e.g., polyphenols), including those known from grape seed, licorice, and oregano, has been observed in previous cell-based in vitro studies (Kim et al., 2012; Pinent et al., 2016; Zou et al., 2016a). Also this study confirms a significant induction of ROS after stimulation with H_2_O_2_, and this induction was significantly attenuated by pretreatment of the cells with D-DC, GRS, LIC, and ORE. In contrast, the PLA, OAK, MENT, or MES did not show a significant influence at the tested concentrations.

In addition to ROS scavenging effects, activities of phytogenic substances on regulation of the redox sensitive transcription factor nuclear factor erythroid 2-related factor (**Nrf2**) and its cytoprotective target genes have been described (Scapagnini et al., 2011; Cheng et al., 2013; Gessner et al., 2017). In this study, we observed a very strong basal expression level of antioxidative genes in IPEC-J2. Stimulation with H_2_O_2_ with or without test substance treatment did not significantly influence these levels, with the exception of HMOX1, which was significantly increased by stimulation of the cells with H_2_O_2_, but no significant effect of phytogenic-treatment has been observed. The strong basal expression of antioxidative genes might result from the specific cultivation conditions in vitro, as cultured cells are exposed to relatively high levels of oxygen and ROS (Halliwell, 2014). However, in previous studies, a sequential expression of antioxidative target genes after prolonged incubation with H_2_O_2_ or phytogenic compounds up to 24 h has been shown (Jin et al., 2016; Zou et al., 2016a). A prolonged stimulation with H_2_O_2_ and the phytogenic substances may be required to overwhelm the strong basal antioxidant capacity of IPEC-J2 and to reveal the potential influence of phytogenic compounds. In our study, the ROS scavenging activity of the phytogenic substances rather than the activation of the antioxidative defense system seemed to be the main antioxidative mechanism responsible for oxidative stress reduction.

Oxidative stress is more than a threat to animal health in its own right; it is also directly associated with the induction of intestinal inflammation (Gessner et al., 2017). ROS play a central role in the induction of the proinflammatory transcription factor NF-κB, which is one of the master regulators of innate and adaptive immune reactions. Upon activation of NF-κB by ROS or other stimuli including cytokines, bacterial components such as lipopolysaccharide (LPS), and other causative agents, a long cascade of inflammatory molecules (e.g., proinflammatory cytokines, chemokines, and adhesion molecules) is activated (Pantano et al., 2006). Inflammation induced by weaning or bacterial challenges in piglets causes significant changes in gut morphology, enhances intestinal permeability, and is directly linked to poor animal performance and impaired health (Pié et al., 2004; Xiao et al., 2016; Gessner et al., 2017). In vivo studies on different essential oils and plant extracts in piglet feed showed inflammation-restricting properties of these PFA, along with beneficial effects on gut morphology and animal growth performance (Kroismayr et al., 2008; Li et al., 2012; Gessner et al., 2013). Therefore, we analyzed the effects of the PFA D-DC and its individual phytogenic components on the regulation of the NF-κB signaling pathway in a challenged porcine cell culture model.

Stimulation of IPEC-J2 with TNF-α effectively induced an inflammatory response, as indicated by a strong increase of NF-κB activation, whereas pretreatment of the cells with D-DC and ORE had a significant inhibitory effect. The findings for ORE confirm the results of in vivo studies, in which ORE has been shown to lower the activity of NF-κB in the jejunum of piglets (Kroismayr et al., 2008; Zou et al., 2016b). MENT did not significantly reduce NF-κB activity and, surprisingly, also MES did not affect NF-κB activity at all, although this effect has been described for some of its derivatives (Zhang et al., 2012). As a broader concentration range of MES was investigated in the prior study, we assume that MES might affect the inflammatory response in a different concentration range. Interestingly, PLA demonstrated anti-inflammatory activity at 10 and 20 µg/mL, but up-regulated NF-κB at 40 µg/mL. Unexpected results were also observed for GRS, LIC, and OAK. Although these substances did not affect NF-κB activation without TNF-α stimulation, they demonstrated a proinflammatory effect upon stimulation of IPEC-J2 with TNF-α. As these potential proinflammatory effects have not been verified via ELISA and RT-qPCR analysis, we hypothesize that they are possibly caused by the formation of artifacts—a phenomenon, which has been seen in reporter gene assays before (Auld et al., 2008).

The anti-inflammatory activities of D-DC and ORE have been further shown via analysis of the prominent NF-κB target genes IL-6, CXCL8, TNF-α, and CCL2 on mRNA level (RT-qPCR) and IL-6 on protein level (ELISA). Both substances effectively inhibited the expression of IL-6, CXCL8, and CCL2 and significantly reduced the release of IL-6. GRS treatment was associated with lower expression of IL-6 and TNF-α at the mRNA level, but not at protein level. None of the other test substances affected the inflammatory parameters at the mRNA level or the protein level at the chosen test concentrations. In contrast to our study, inflammation-restricting properties of a grape seed extract have been shown in vitro and in vivo (Gessner et al., 2012; Gessner et al., 2013). The divergent result may be explained by different test concentrations used in this study. The results obtained from testing D-DC and ORE support the hypothesis that PFA act through modulation of the NF-κB pathway and related target genes.

AGP are usually added to animal feed at subtherapeutic levels to improve the growth performance (Van Lunen, 2003). Different mechanisms have been claimed to be responsible for the growth promoting effects including metabolic effects, nutritional effects, or effects mediated by interference with the microbiota (Cromwell, 2002; Gadde et al., 2017). It is further hypothesized that the growth promoting effect is based on inflammation-restricting characteristics of the AGP (Niewold, 2007). Although an anti-inflammatory activity of various AGP is corroborated by several authors (Kroismayr et al., 2008; Khadem et al., 2014; Palamidi et al., 2016), the opinions still differ regarding their real mode of action (Broom, 2017). Because there is evidence of anti-inflammatory activity of several macrolides (e.g., tylvalosin, tilmicosin, and TYL) on immune cells (Cao et al., 2006; Buret, 2010; Zhao et al., 2014), we included the APG TYL in tests on NF-κB and the inflammatory target genes. Treatment of IPEC-J2 with TYL at concentrations commonly used in feed did not affect the activity of NF-κB or any of the studied target genes. Thus, it might act on growth performance by a different mechanism or require a different experimental design.

In conclusion, we show potential benefits of the PFA D-DC and selected phytogenic components in the regulation of the intestinal immune response and cellular redox homeostasis in the IPEC-J2 cell culture model. These findings provide more accurate knowledge of the mechanism of action of PFA used in animal nutrition, and they contribute to a better understanding of the benefits of using PFA in livestock production. Additional studies on the influence of the tested phytogenic substances on antioxidative and anti-inflammatory parameters in vivo are recommended.
